# The Safety and Efficacy of the Mono‐Bi CrossLIFT Technique Utilizing Capacitive‐Coupled Sequential Monopolar and Bipolar Pulsed Radiofrequency for Simultaneous Facial Skin Tightening and Contouring: A Clinical Case Series

**DOI:** 10.1111/jocd.70504

**Published:** 2025-10-15

**Authors:** Kentaro Oku

**Affiliations:** ^1^ HILLS GRACE CLINIC Yokohama Japan

**Keywords:** facial contouring, facial rejuvenation, Mono‐Bi CrossLIFT Technique, radiofrequency (RF), sequential monopolar–bipolar radiofrequency (SMBPRF), skin tightening

## Abstract

**Background:**

Noninvasive facial rejuvenation techniques that emphasize skin tightening and contouring continue to gain popularity due to their minimally invasive nature and favorable clinical outcomes. Recent technological advancements, such as capacitive‐coupled sequential monopolar and bipolar pulsed radiofrequency (SMBPRF), enable simultaneous targeted collagen remodeling within the dermis and contraction of subdermal fibrous tissues.

**Objective:**

To comprehensively evaluate the clinical safety and efficacy of the Mono‐Bi CrossLIFT Technique utilizing SMBPRF for simultaneous facial skin tightening and contour improvement.

**Methods:**

Sixteen healthy adults (aged 36–59 years, Fitzpatrick skin types II–IV) with mild to moderate facial skin laxity and sagging were enrolled in this study. Each participant underwent a single treatment session of SMBPRF using the Mono‐Bi CrossLIFT Technique. Quantitative assessments, including skin tightening distances (mm) and changes in lower facial tissue thickness (mm), were performed at baseline and 24 weeks post‐treatment using high‐resolution three‐dimensional imaging. Clinical efficacy was evaluated using the Global Aesthetic Improvement Scale (GAIS) by both an independent dermatologist and the subjects themselves. Pain levels and adverse events were also recorded throughout the study period.

**Results:**

All 16 subjects completed the study. Statistically significant improvements in both skin tightening and facial contouring were observed in 15 subjects. The mean skin tightening distance was 0.82 ± 0.36 mm (*p* = 3.528 × 10^−7^), and the mean reduction in tissue thickness was 0.89 ± 0.40 mm (*p* = 6.704 × 10^−7^). According to the Global Aesthetic Improvement Scale (GAIS), 15 subjects were rated as “Improved” or better in both objective and subjective assessments. The mean procedural pain score was 1.4 ± 0.6 on a 5‐point scale. No severe adverse events were reported during the treatment or the follow‐up period.

**Conclusion:**

Mono‐Bi CrossLIFT Technique utilizing SMBPRF demonstrated significant safety and bidirectional improvement, supporting its clinical utility as a noninvasive method for comprehensive facial rejuvenation.

## 1 Introduction

Facial aging involves complex physiological changes, including decreased synthesis of collagen and elastin, cellular senescence, and structural weakening of supporting ligaments, all of which contribute to visible skin laxity, sagging, and wrinkle formation. Noninvasive rejuvenation modalities, particularly those utilizing radiofrequency (RF), have gained popularity as alternatives to invasive surgical procedures due to their ability to induce targeted collagen remodeling with minimal downtime [1, 2].

Traditional RF treatments predominantly employ either monopolar or bipolar RF individually. Monopolar RF provides deep tissue heating, making it suitable for substantial collagen contraction in deeper dermal and subdermal layers [3]. Conversely, bipolar RF predominantly targets superficial dermal layers, enhancing skin texture and reducing fine wrinkles [4]. However, single‐modality RF treatments often fail to simultaneously achieve effective facial skin tightening and contouring, necessitating sequential or combined approaches.

Recent technological advances have introduced capacitive‐coupled sequential monopolar–bipolar pulsed radiofrequency (SMBPRF), which sequentially utilizes monopolar and bipolar energy within a single RF application. This sequential delivery optimizes collagen remodeling across multiple tissue layers: initial capacitive‐coupled monopolar RF sub‐pulses heat subdermal fibrous tissues, reducing tissue impedance and thereby enhancing the subsequent efficacy of capacitive‐coupled bipolar RF sub‐pulse delivered to superficial layers [5].

Monopolar RF delivers energy vertically, penetrating deeply from the skin surface toward underlying structures, whereas bipolar RF applies energy horizontally, parallel to the skin surface. To maximize the therapeutic benefits from this directional distinction, vertical energy should be strategically utilized for deep tissue “vertical contraction,” while horizontal energy targets superficial dermal fibrous tissues for “horizontal contraction.” Leveraging this axial directionality of RF energy, we developed the Mono‐Bi CrossLIFT Technique using SMBPRF.

The Mono‐Bi CrossLIFT Technique capitalizes on this sequential RF energy approach, delivering capacitive‐coupled monopolar and bipolar RF along targeted vectors to achieve optimal simultaneous facial skin tightening and contouring. The aim of this study is to evaluate the clinical efficacy and safety of the Mono‐Bi CrossLIFT Technique utilizing SMBPRF.

## 2 Materials and Methods

### 2.1 Ethics Approval

The study protocol was reviewed and approved by the Shiba Palace Clinic Institutional Review Board (approval number 159980_rn‐40 962). The study was conducted in strict accordance with the ethical principles outlined in the Declaration of Helsinki (1975). Prior to enrollment, comprehensive written informed consent was thoroughly obtained from each participant after providing detailed verbal and written explanations about the study objectives, treatment procedures, potential risks, benefits, and alternatives.

### 2.2 Study Plan

The study period spanned from August 2023 to March 2025 and involved two structured visits. The initial baseline visit included a detailed clinical evaluation, photographic documentation, and informed consent procedures, followed immediately by a single treatment session. The follow‐up visit occurred 24 weeks post‐treatment, during which clinical outcomes were comprehensively evaluated.

### 2.3 Inclusion and Exclusion Criteria

Subjects included healthy adults aged 36–75 years who presented with visible signs of facial aging and were seeking skin‐tightening treatments. Exclusion criteria comprised the presence of any electrical implants (e.g., pacemakers, defibrillators), permanent implants in the treatment area (e.g., metal plates, silicone implants, screws), current or prior history of skin cancer, immunocompromised conditions, history of skin disorders such as keloids or impaired wound healing, skin treatments (filler injections, microneedling, or laser therapy) within the previous 6 months, or recent isotretinoin use.

### 2.4 Devices Used

**FIGURE 1:**
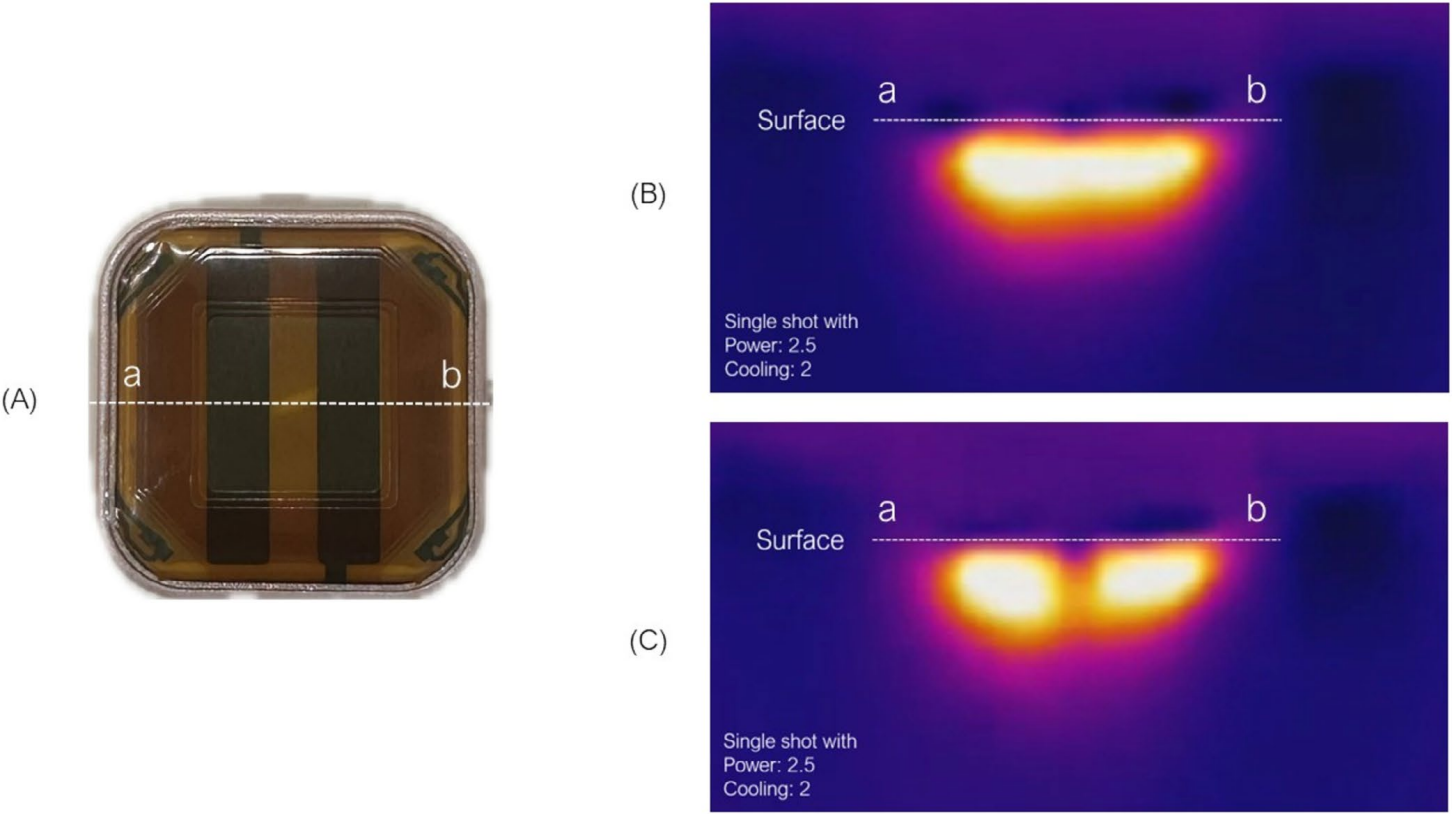
(A) Transducer surface of “High tip” of DENSITY. The two dark bands indicate the electrodes. (B) Sagittal thermographic image illustrating the thermal zone during bipolar RF subpulse irradiation using the high tip shown in (A). (C) Sagittal thermographic image illustrating the thermal zone during monopolar RF subpulse irradiation using the high tip shown in (A).

A capacitive‐coupled sequential monopolar–bipolar pulsed radiofrequency (SMBPRF) device DENSITY (Jeisys Medical Inc.) was utilized for all subjects. The transducer surface of the DENSITY device (“High tip”) is illustrated in Figure 1A. During RF irradiation, thermal zones are generated as shown in Figure 1B,C. The device sequentially delivered monopolar and bipolar RF energy, initially employing monopolar RF sub‐pulses to achieve bulk heating of deeper tissues, followed by bipolar RF sub‐pulse targeting superficial dermal tissues.

### 2.5 Intervention

Treatment preparation involved meticulous cleansing of the facial skin to thoroughly remove makeup and skincare products. After visually confirming the complete removal of any residual substances from the skin, coupling fluid was applied evenly onto the treatment area, and SMBPRF was administered to the entire face and submental region using the Mono‐Bi CrossLIFT Technique.

Mono‐Bi CrossLIFT Technique consists of a total of six strategic passes, alternating between vertically oriented monopolar RF passes targeting subdermal retinacular cutis and fibrous septa and horizontally oriented bipolar RF passes targeting dermal tissue.

#### 2.5.1 Passes 1, 3, and 5 for Horizontal Changes (Figure 2)

**FIGURE 2:**
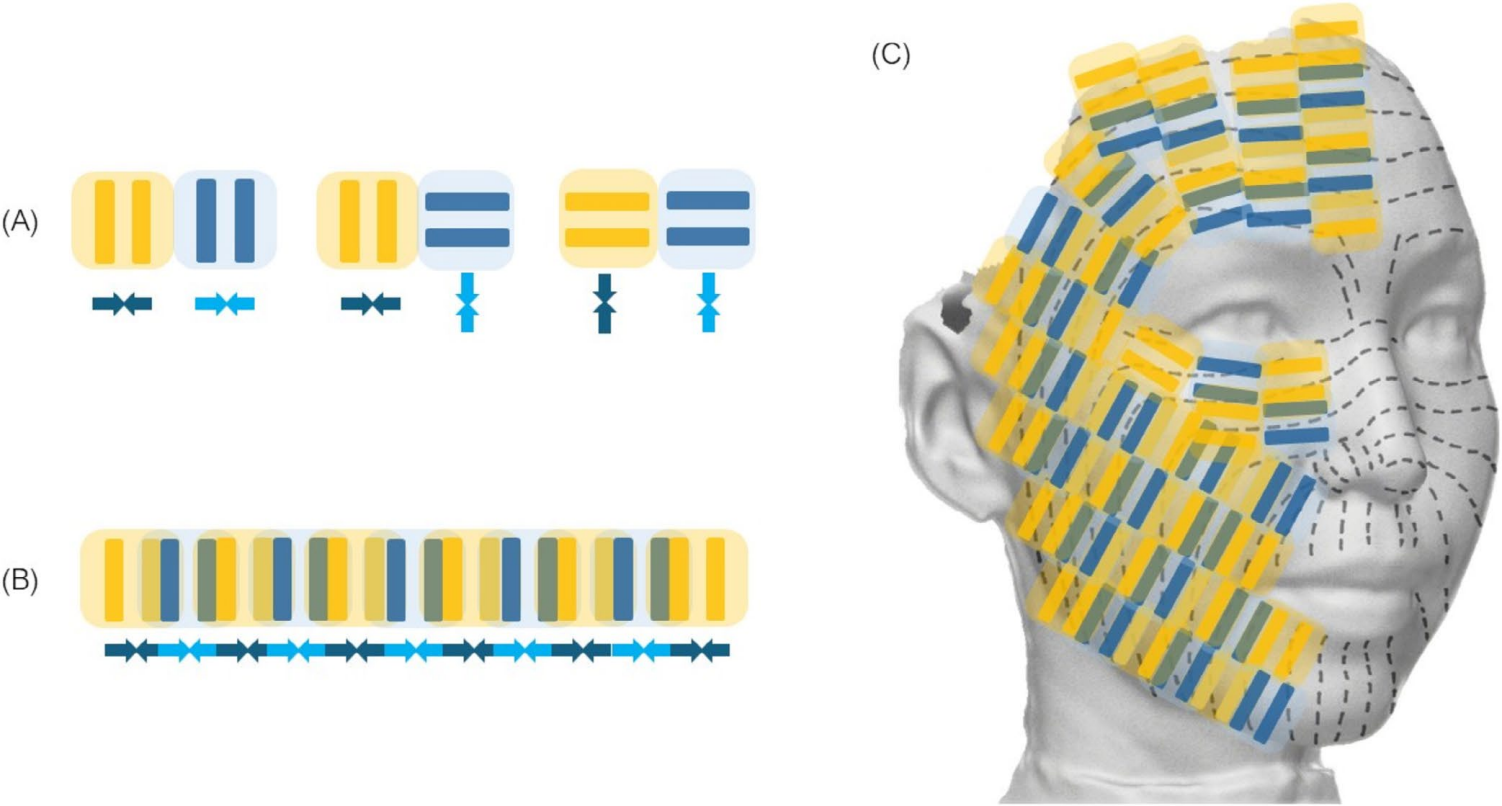
(A) Schematic illustrations of transducers (yellow and blue) and directions of collagen contraction induced by thermal zones (arrows). When RF shots are delivered as shown on the left, the contraction direction aligns correctly, but the spacing between the first and second shots reduces contraction efficiency. The middle configuration demonstrates misalignment in contraction directions, leading to decreased efficiency. On the right, although the direction is appropriate, the inability to interconnect thermal zones also diminishes contraction efficiency. (B) Schematic representation of the optimal irradiation method, considering the thermal zone of bipolar RF, designed to maximize tissue contraction efficiency. (C) Illustration of the irradiation method for the right half of the face. The dashed lines represent RSTL. RF irradiation is sequentially overlapped to ensure the thermal zones are applied perpendicularly to the direction of the RSTL, optimizing thermal efficacy.

Facial wrinkles and sagging typically develop along relaxed skin tension lines (RSTL). These lines are formed perpendicular to the direction in which the skin is persistently stretched by long‐term factors such as gravity, muscular contractions, external stimuli, and aging. Therefore, to maximize the horizontal contraction effects, it is essential to avoid inappropriate irradiation methods lacking continuity in thermal zones, as depicted in Figure 2A. After delivering the initial RF shot, the handpiece is horizontally repositioned by 11 mm, corresponding to the width of the thermal zone created by bipolar RF, and the subsequent shot is administered, as depicted in Figure 2B. Furthermore, as shown in Figure 2C, aligning the thermal zones perpendicular to the RSTL maximizes the efficiency of thermal energy delivery. This strategic irradiation method creates thermal zones oriented to achieve an overall upward and diagonal lifting effect.

#### 2.5.2 Passes 2, 4, and 6 for Vertical Changes (Figure 3)

**FIGURE 3:**
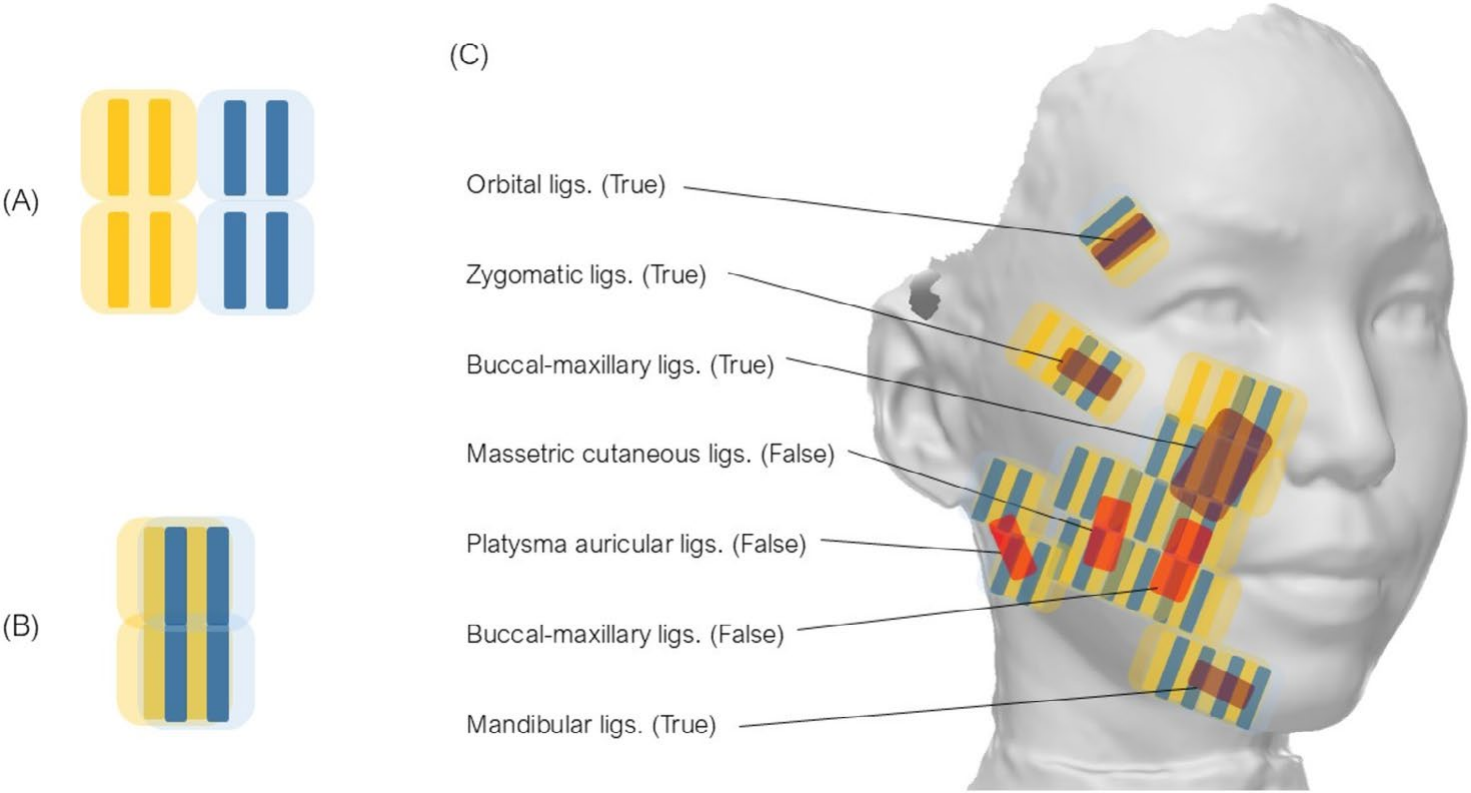
(A) Schematic illustration showing RF irradiation without overlapping treatment areas. (B) Schematic illustration showing optimal irradiation technique with monopolar RF to achieve densely overlapped thermal zones. (C) Schematic illustration demonstrating the locations of true and false retaining ligaments in the right half of the face and the irradiation technique to densely target these ligamentous structures with thermal zones.

The thermal zones created by capacitive‐coupled monopolar RF form two rectangular prisms extending vertically from the electrodes toward the underlying bone structure (Figure 1C). As illustrated by the standard irradiation protocol shown in Figure 3A, delivering RF shots without overlapping results in sparse thermal coverage, making it difficult to achieve sufficient deep tissue heating. To optimize thermal efficiency, after each RF shot, the handpiece is horizontally repositioned by 4 mm, corresponding to the distance between the electrodes, and subsequently shifted perpendicularly by 11 mm, equivalent to the width of the handpiece, as illustrated in Figure 3B. This sequential movement ensures optimal overlapping and comprehensive coverage of the targeted treatment area. Additionally, to maximize the deep thermal effects of monopolar RF, treatment passes are strategically delivered to areas densely populated with retinacular cutis derived from retaining ligaments, thereby promoting vertical shortening of the distance between the skin surface and underlying bone, as illustrated in Figure 3C.

Energy parameters ranged from 1.0 to 3.0 and were adjusted individually according to patient‐reported pain levels throughout the treatment session. Energy settings were optimized to produce noticeable warmth without significant discomfort, following the established guidelines and protocols from previous studies [6, 7]. The DENSITY device incorporates an adjustable cooling system; however, it has been demonstrated that temperature elevation at the targeted monopolar RF treatment depth (approximately 4.0 mm) remains unaffected by changes in cooling level. Therefore, relatively higher RF energy levels can be safely applied in conjunction with increased cooling settings to enhance patient comfort.

Each treatment session consisted of approximately 500–600 RF shots, systematically applied to cover the entire facial and submental regions. The total thermal energy delivered per session ranged from approximately 40 to 60 kJ.

### 2.6 Objective Evaluation

Pre‐ and post‐treatment evaluations were performed using high‐resolution 3D imaging generated from clinical photographs taken from three different angles with the Vectra H2 system (Canfield Scientific Inc.), enabling accurate quantification of both skin tightening distance (horizontal change) and changes in tissue thickness (vertical change) at baseline and 24 weeks post‐treatment. Photographs were consistently obtained by the same independent operator, who was not involved in the treatment procedure. Patients were strictly instructed to maintain neutral facial expressions during image capture to ensure consistency between assessments.

Considering common concerns of patients seeking RF treatment—such as facial sagging around the perioral area and prominent nasolabial folds (NLF)—the following analytical methods were employed to evaluate pre‐ and post‐treatment 3D images: For horizontal changes, a baseline axis was defined as the line connecting the oral commissure to the external auditory canal. Vectors identified by the imaging system within ±22.5° of this baseline axis were considered relevant for assessing horizontal skin tightening, consistent with previously published methods [8]. The longest vector within this defined angular range was measured, with baseline defined as 0 mm. For vertical changes, the lower face was defined as the region below the line extending from the nasal ala to the external auditory canal, to comprehensively evaluate areas containing a high density of retinacular cutis derived from true and false retaining ligaments. Within this defined region, the point demonstrating the greatest negative vertical change was identified and measured, with baseline defined as 0 mm.

Additionally, clinical outcomes were objectively assessed by an independent dermatologist using the Global Aesthetic Improvement Scale (GAIS), which rates improvements on a 5‐point scale: very much improved, much improved, improved, no change, or worsened.

### 2.7 Subjective Evaluation

Procedural pain experienced by subjects during treatment was assessed using a validated 5‐point Likert scale (1: no pain, 2: mild pain, 3: moderate pain, 4: severe pain, 5: very severe pain). Additionally, treatment satisfaction was subjectively evaluated by each participant using the same GAIS employed in the objective evaluation.

### 2.8 Safety

Any side effects or complications related to the procedure were meticulously documented by the dermatologist throughout the treatment and follow‐up periods.

### 2.9 Statistical Analysis

A one‐sample *t*‐test was employed to evaluate horizontal and vertical changes. Statistical significance was set at *p* < 0.05. Continuous variables were presented as mean ± standard deviation.

## 3 Results

All 16 participants completed the trial.

### 3.1 Demographics

Sixteen Japanese subjects, comprising one male and 15 females (mean age 49.4 ± 6.1 years) with Fitzpatrick skin types II (*n* = 2), III (*n* = 11) and IV (*n* = 3), were included in this study.

### 3.2 Quantitative Analysis

**FIGURE 4:**
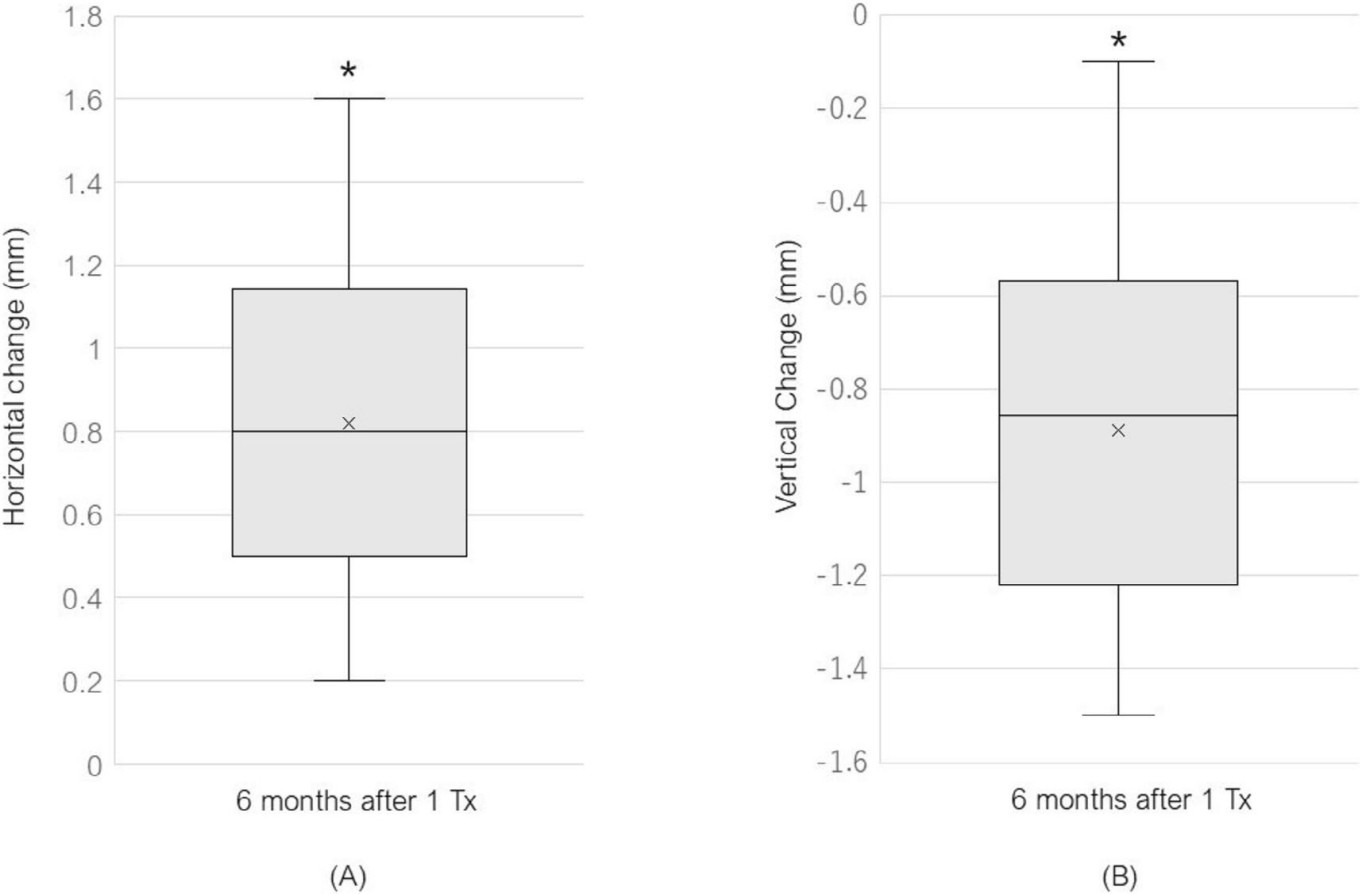
(A) Box‐and‐whisker plot showing horizontal changes at 6 months post‐treatment relative to baseline (defined as 0 mm). *Statistical significance (*p* < 0.05). (B) Box‐and‐whisker plot illustrating vertical changes at 6 months post‐treatment relative to baseline (defined as 0 mm). *Statistical significance (*p* < 0.05).

The measurement data revealed a statistically significant mean horizontal change of 0.82 ± 0.34 mm (*p* = 3.528 × 10^−7^ < 0.05) at 24 weeks post‐treatment, with a maximum observed change of 1.6 mm, indicating clinically meaningful improvement in skin tightening (Figure 4A). Similarly, significant vertical changes were observed, with a mean change of −0.89 ± 0.40 mm (*p* = 6.704 × 10^−7^ < 0.05) and a maximum observed change of −1.5 mm, demonstrating substantial improvement in facial contouring (Figure 4B).

### 3.3 Clinical Outcomes

**FIGURE 5:**
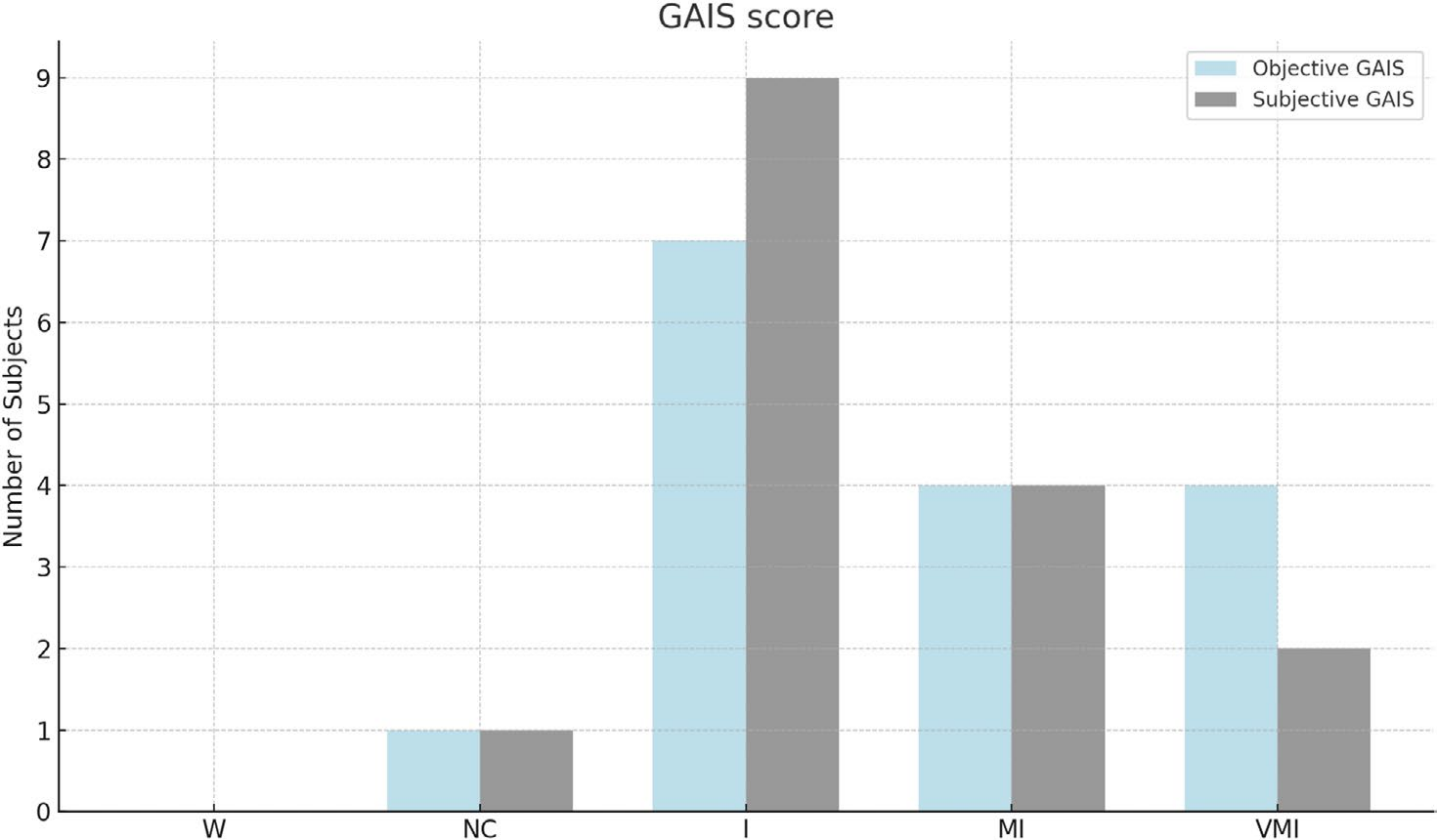
Objective (blue) and subjective (gray) GAIS evaluations. I, improved; MI, much improved; NC, no change; VMI, very much improved; W, worsened.

Objective GAIS assessments showed the following distribution: very much improved (VMI; *n* = 4, 25%), much improved (MI; *n* = 4, 25%), improved (I; *n* = 7, 43.8%), no change (NC; *n* = 1, 6.3%), and worsened (W; *n* = 0, 0%). Subjective GAIS evaluations by participants indicated: very much improved (VMI; *n* = 2, 12.5%), much improved (MI; *n* = 4, 25%), improved (I; *n* = 9, 56.3%), no change (NC; *n* = 1, 6.3%), and worsened (W; *n* = 0, 0%). The distribution of these scores is illustrated in Figure 5.

**FIGURE 6:**
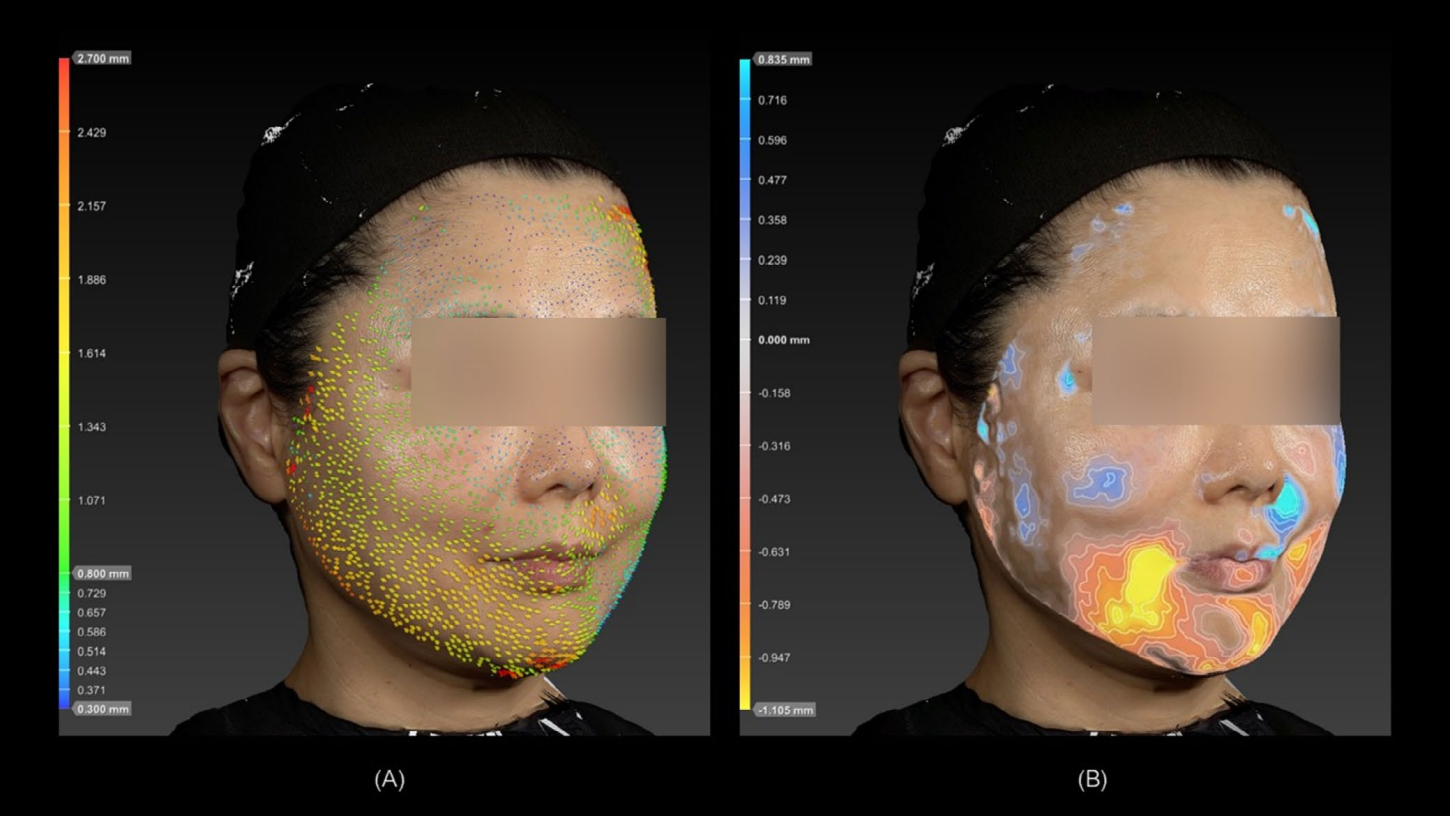
A 54‐year‐old female, Fitzpatrick skin type III. (A) Noticeable skin tightening occurred approximately parallel to the baseline axis connecting the oral commissure to the external auditory canal. The maximum horizontal change within the defined angular range was 1.6 mm. (B) Noticeable negative changes (the regions indicated in orange to yellow) occurred around the marionette lines in the lower face region (defined as the area below the line connecting the nasal ala to the external auditory canal). The maximum observed vertical change was −1.1 mm.

**FIGURE 7:**
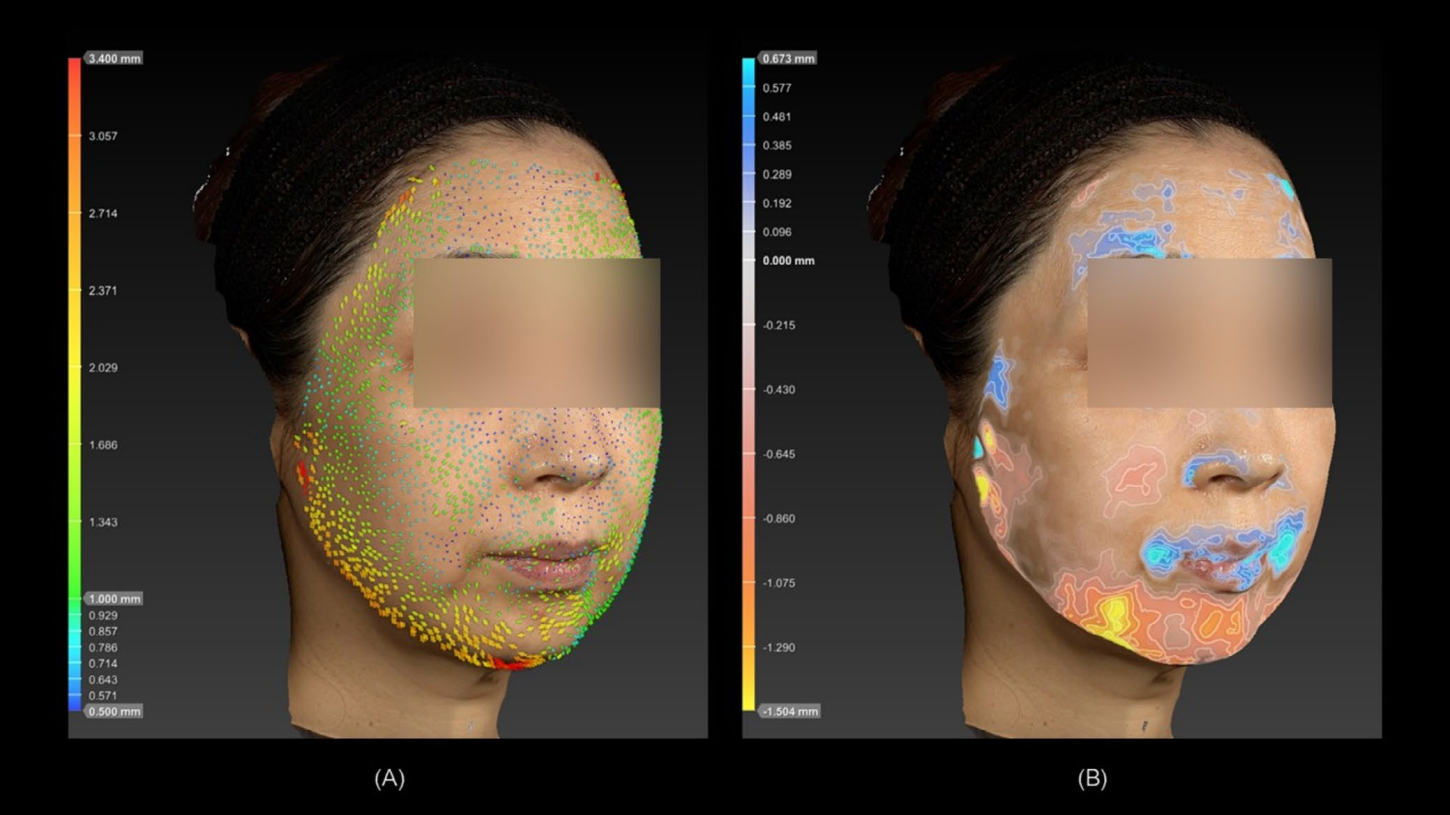
A 59‐year‐old female, Fitzpatrick skin type III. (A) Prominent skin tightening was observed around the jawline region. The effective horizontal change within the defined angular range was 1.3 mm. (B) Significant negative vertical changes were observed around the marionette lines and the platysma auricular ligament region. The maximum vertical change was −1.5 mm.

Representative clinical cases demonstrating pre‐ and post‐treatment changes in horizontal (skin tightening) and vertical (facial contouring) dimensions are shown in Figures 6 and 7.

### 3.4 Complications

Participants reported minimal procedural discomfort, with an average pain score of 1.4 ± 0.6 of 5.

No adverse events such as burns, prolonged erythema, or scarring were observed throughout the treatment and follow‐up periods. All subjects resumed routine activities immediately after treatment, underscoring the minimal recovery time associated with this procedure.

## 4 Discussion

Radiofrequency (RF) therapy has a well‐established history in aesthetic medicine and is widely recognized for its noninvasive nature and consistent clinical efficacy. Over the past two decades, RF treatments have been effectively utilized for various indications, including facial tightening [9, 10, 11, 12, 13], improvements in skin texture [14], melasma [15], and pore size reduction [16]. Numerous studies have demonstrated significant clinical outcomes, highlighting the ability of RF to induce controlled thermal injury within dermal and subdermal tissues, thereby stimulating collagen synthesis and remodeling. In this study, improvements in facial tightening and facial contouring were also observed, consistent with previous reports.

Despite extensive literature on RF applications, to our knowledge, no previous studies have simultaneously evaluated both vertical and horizontal changes induced exclusively by RF treatments in facial tissues based on measurement results. Thus, our study using the Mono‐Bi CrossLIFT Technique with capacitive‐coupled sequential monopolar and bipolar pulsed radiofrequency (SMBPRF) represents the first clinical investigation of simultaneous noninvasive assessment of vertical and horizontal facial contractions.

The effectiveness of combined monopolar and bipolar RF observed in this study can be explained by previously reported mechanisms involving impedance changes and vertical energy delivery. According to Oh et al., monopolar RF effectively heats deeper tissue layers, subsequently reducing tissue impedance [5]. This impedance reduction enhances subsequent bipolar RF energy delivery. Since tissue electrical conductivity increases with temperature, preheating tissues with monopolar RF optimally prepares the tissue to receive bipolar RF energy more efficiently. Furthermore, RF energy preferentially concentrates in areas with reduced impedance. Thus, the initial vertical deep heating provided by capacitive‐coupled monopolar RF enhances the efficacy of subsequent capacitive‐coupled bipolar RF in targeting deeper dermal layers rich in fibroblasts. These described mechanisms align well with the significant clinical effectiveness of SMBPRF demonstrated in the current study.

Regarding facial volume, this can be conceptualized as a product of surface area and depth. In facial anatomy, surface area corresponds to the skin surface, whereas depth relates to the length of retinacular cutis and fibrous septa beneath the skin. Monopolar RF preferentially heats and remodels collagen fibers, including retinacular cutis and fibrous septa, effectively reducing subdermal depth and thereby contributing to overall volume reduction. To optimize volume reduction, simultaneous remodeling of dermal tissue through bipolar RF, in conjunction with monopolar RF, is beneficial. In three‐dimensional anatomical terms, the *Z*‐axis represents facial depth, and the XY‐plane represents facial surface area. Monopolar RF reduces volume by negatively impacting depth along the *Z*‐axis, while bipolar RF contributes to volume reduction by reducing the XY‐plane surface area. The direction of bipolar RF energy application is crucial. Assuming relaxed skin tension lines (RSTL) align parallel to the *X*‐axis, collagen fibers are predominantly elongated along the *Y*‐axis. Delivering thermal energy preferentially along the *Y*‐axis thus maximizes collagen contraction and improves clinical outcomes. Mono‐Bi CrossLIFT Technique explicitly determines electrode orientation to optimize these directional energy applications.

Although the results of this study are promising, several limitations must be explicitly acknowledged. First, the small sample size restricts the generalizability of the findings. A larger cohort would allow for more robust statistical analysis and more definitive conclusions regarding the clinical efficacy of the Mono‐Bi CrossLIFT Technique with SMBPRF. Second, the short follow‐up period limits the ability to assess long‐term durability and sustainability of simultaneous facial skin tightening and contouring effects. Extended follow‐up studies would provide crucial insights into the longevity of clinical outcomes and potential late‐onset complications. Additionally, the study cohort was relatively homogeneous, and broader demographic representation—including diverse age ranges, skin types, and ethnicities—would enhance the external validity and applicability of these findings to a wider patient population.

Considering these limitations, implications for future clinical practice and research include the necessity of conducting randomized controlled trials with larger sample sizes and prolonged follow‐up periods to validate the safety and efficacy of SMBPRF. Such studies would contribute significantly to optimizing energy delivery parameters, refining patient selection criteria, and potentially expanding clinical indications. Further research should also explore combination treatments, assessing synergistic effects with other aesthetic modalities, thereby potentially enhancing therapeutic outcomes. Moreover, standardized protocols for objective measurement of facial tightening and volumetric changes using advanced imaging techniques could be developed to facilitate comparability across future studies and enhance clinical decision‐making.

## 5 Conclusion

The Mono‐Bi CrossLIFT Technique employing capacitive‐coupled sequential monopolar and bipolar pulsed RF demonstrates high safety and efficacy in achieving significant facial skin tightening and contour improvement simultaneously, with minimal discomfort and no notable adverse events. These clinically proven results strongly support its role as a reliable and effective noninvasive solution for comprehensive facial rejuvenation, emphasizing both patient safety and high treatment effectiveness. Further investigation is encouraged to confirm long‐term outcomes and optimize clinical protocols.

## Author Contributions

Kentaro Oku was solely responsible for all aspects of this study, including the conceptualization and study design, acquisition and interpretation of data, drafting and critically revising the manuscript, and approval of the final version. The author agrees to be accountable for all aspects of the work, ensuring that any questions related to the accuracy or integrity of the content are appropriately investigated and resolved.

## Ethics Statement

The study protocol was reviewed and approved by the Shiba Palace Clinic Institutional Review Board (approval number 159980_rn‐40 962). The study was conducted in strict accordance with the ethical principles outlined in the Declaration of Helsinki (1975). All participants provided written informed consent prior to enrollment.

## Conflicts of Interest

The author declares no conflicts of interest.

## Data Availability

The data that support the findings of this study are available from the corresponding author upon reasonable request.
